# P056 Recurrent strokes in a young man–a missed opportunity?

**DOI:** 10.1093/rap/rkag113.086

**Published:** 2026-11-03

**Authors:** Sajeel Ahmed, Kenzie Maginnis, David Coady

**Affiliations:** South Tyneside and Sunderland NHS Foundation Trust, Sunderland, United Kingdom; South Tyneside and Sunderland NHS Foundation Trust, Sunderland, United Kingdom; South Tyneside and Sunderland NHS Foundation Trust, Sunderland, United Kingdom

## Abstract

**Introduction:**

Takayasu arteritis is a rare large-vessel vasculitis affecting the aorta and its major branches. Although cerebrovascular complications are recognised, stroke is an uncommon presenting manifestation and may delay diagnosis, particularly in the absence of systemic symptoms. We present a 27-year-old man who experienced recurrent ischaemic strokes over a two-year period before Takayasu arteritis was diagnosed. Despite progressive cerebrovascular disease, inflammatory markers remained normal throughout and constitutional symptoms were absent. This case highlights the diagnostic challenges of atypical Takayasu arteritis and emphasises the importance of considering large-vessel vasculitis in young patients with unexplained recurrent stroke.

**Case description:**

A 27-year-old male smoker (10 cigarettes/day) with no significant medical history presented in January 2021 with progressive disorientation, dizziness, visual disturbance and unsteadiness. Neuroimaging demonstrated a right temporo-occipital infarct in the posterior cerebral artery territory. MRI also identified previous occipital and lacunar infarcts, whilst MR angiography showed severe bilateral internal carotid artery stenosis. He was commenced on antiplatelet and statin therapy.

Six months later he re-presented with worsening balance and visual symptoms. Examination demonstrated a marked left internuclear ophthalmoplegia with horizontal nystagmus. MRI confirmed acute pontine and midbrain infarcts. Repeat angiography demonstrated progression of vasculopathy, including obstruction of both internal carotid and posterior cerebral arteries, loss of flow within the left middle cerebral artery and probable right vertebral artery stricturing. Dual antiplatelet therapy was initiated.

In October 2021, weak lower limb pulses and intermittent claudication after approximately 50 metres were identified, raising suspicion of systemic vascular disease. PET-CT at the time showed no active uptake and extensive thrombophilia screen was reassuring.

In January 2023 he developed dysarthria and worsening right-sided weakness. MRI demonstrated a new left medullary infarct. Examination revealed dysarthria, horizontal gaze nystagmus and significant functional impairment requiring assistance with activities of daily living. MRI and CT angiography demonstrated ongoing severe vasculopathy. A repeat PET-CT subsequently revealed increased FDG uptake within the femoral arteries, supporting active large-vessel inflammation.

Following multidisciplinary review, a diagnosis of Takayasu arteritis was established. Methotrexate and adalimumab were commenced in July 2023. Since initiation of immunosuppressive therapy, no further cerebral infarcts have occurred. Residual deficits include right-sided weakness, dysarthria, left homonymous hemianopia and reduced mobility requiring an electric wheelchair.

**Discussion:**

This case illustrates an atypical presentation of Takayasu arteritis and the consequences of delayed diagnosis. The patient experienced three ischaemic strokes over two years before a unifying diagnosis was reached. Although stroke is a recognised complication of Takayasu arteritis, it more commonly occurs following established disease and is rarely the dominant presenting feature.

Several factors contributed to diagnostic delay. The patient lacked constitutional symptoms typically associated with large-vessel vasculitis, including fever, weight loss and malaise. Inflammatory markers remained normal despite progressive vascular inflammation and ongoing cerebrovascular injury.

Progressive intracranial stenosis initially prompted consideration of alternative diagnoses including moyamoya disease, primary angiitis of the CNS, primary CNS vasculitis and inherited vasculopathies. However, the subsequent development of diminished lower limb pulses and intermittent claudication raised suspicion for systemic large-vessel disease. The vascular distribution was also atypical. Whilst severe bilateral carotid stenosis was present, PET-CT demonstrated active inflammation within the femoral arteries rather than the more commonly recognised subclavian or aortic territories. These atypical features complicated the diagnosis and ultimately required synthesis of clinical findings with serial vascular and functional imaging before a multidisciplinary diagnosis of Takayasu arteritis was reached.

The case highlights the importance of comprehensive vascular examination and multimodal imaging in young patients with unexplained recurrent stroke. Earlier recognition of large-vessel vasculitis may have altered the disease trajectory and potentially reduced irreversible neurological disability. Following initiation of methotrexate and adalimumab, no further cerebrovascular events have occurred, supporting an inflammatory mechanism underlying disease progression.

This case raises an important question for discussion: in young patients with recurrent stroke, progressive arterial stenosis and limited vascular risk factors, should earlier whole-body vascular assessment and rheumatology involvement be considered even when inflammatory markers are normal and systemic features are absent?

**Key learning points:**

• This case highlighted the importance of maintaining a broad differential diagnosis in young patients with recurrent ischaemic stroke, particularly when the severity of vascular disease appears disproportionate to conventional risk factors. Takayasu arteritis may present with isolated neurological manifestations and can remain clinically active despite normal inflammatory markers and the absence of constitutional symptoms. Consequently, reliance on serological markers alone may contribute to delayed diagnosis.

• A key learning point was the value of repeated clinical assessment. The later development of diminished peripheral pulses and intermittent claudication provided important clues to a systemic vasculopathy and prompted reconsideration of the diagnosis. The case also reinforced the role of multimodal imaging, including serial vascular imaging and PET-CT, in identifying large-vessel inflammation when the diagnosis remains uncertain.

• Perhaps the most important lesson was the potential impact of diagnostic delay. This patient experienced three strokes and sustained significant neurological disability before a diagnosis of Takayasu arteritis was established. Earlier recognition may have facilitated earlier immunosuppressive treatment and potentially reduced irreversible morbidity.

• The principal learning objective from the conference would be to better understand how colleagues investigate young patients with recurrent stroke and progressive arterial stenosis when inflammatory markers are normal and systemic features are absent. In particular, I would be interested in discussing when large-vessel vasculitis should be suspected, the role of PET-CT and other vascular imaging modalities in atypical presentations, and whether earlier rheumatology involvement could shorten diagnostic delays and improve patient outcomes.

